# Biocatalytic membrane: Go far beyond enzyme immobilization

**DOI:** 10.1002/elsc.202000018

**Published:** 2020-06-09

**Authors:** Jianquan Luo, Siqing Song, Hao Zhang, Huiru Zhang, Jinxuan Zhang, Yinhua Wan

**Affiliations:** ^1^ State Key Laboratory of Biochemical Engineering, Institute of Process Engineering Chinese Academy of Sciences Beijing P.R. China; ^2^ School of Chemical Engineering University of Chinese Academy of Sciences Beijing P.R. China

**Keywords:** enzymatic catalysis, enzymatic membrane reactor, membrane fouling, membrane separation, reaction separation integration

## Abstract

Biocatalytic membrane takes advantages of reaction–separation integration as well as enzyme immobilization, which has attracted increasing attentions in online detection and biomanufacturing. However, the high preparation cost, inferior comprehensive performance, and low stability limit its applications. Thus, besides enzyme immobilization, more efforts should be made in biocatalytic membrane configuration design for a specific application to enhance the synergistic effect of reaction and separation and improve its operating stability. This review summarized the recent progress on biocatalytic membrane preparation, discussed different membrane configurations for various applications, finally proposed several challenges and possible solutions, which provided directions and guides for the development and industrialization of biocatalytic membrane.

## Graphical abstract

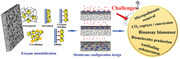

**Abbreviations**
APTES: 3‐aminopropyltriethoxysilane
BPA: bisphenol A
CA: carbonic anhydrase
CNTs: carbon nanotubes
CO_2_: carbon dioxide
GO: graphene oxides
MF: microfiltration
MOFs: metal organic frameworks
NF: nanofiltration
PDA: polydopamine
TA: tannic acid
UF: ultrafiltration

## 1 INTRODUCTION

**FIGURE 1:**
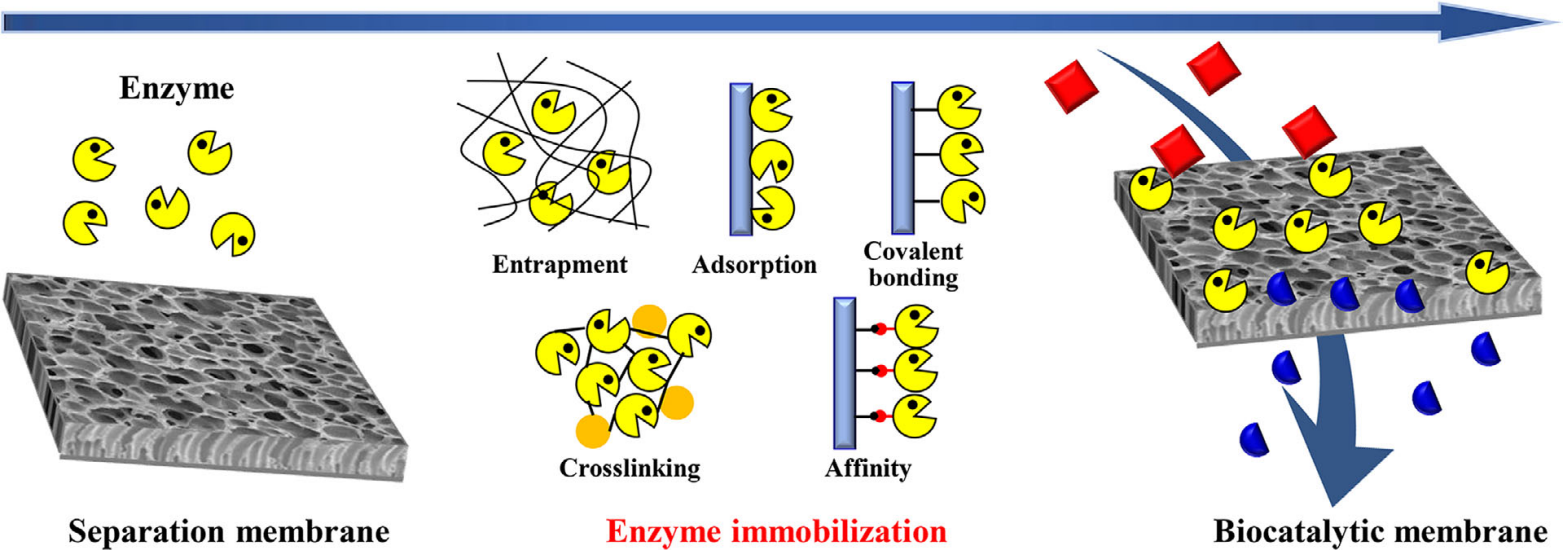
Biocatalytic membrane preparation by various enzyme immobilization techniques

Inspired by structure and functions of cell membrane, biocatalytic membrane is proposed and prepared by immobilizing enzymes in/on a separation membrane (Figure 1) [1]. Using pressure or concentration difference across the membrane as driving force, the enzymatic product can be removed from the reaction system, thus, achieving the integration of membrane separation and enzymatic catalysis. For the enzymatic reaction, due to the timely product removal, biocatalytic membrane can not only reduce product inhibition effect but also avoid side reactions generating byproducts, thus, enhancing the enzymatic conversion efficiency [2]. Moreover, if enzymes are immobilized inside the membrane, the separation function of the membrane would alleviate the burden of enzymatic catalysis and regulate the reaction kinetics [3], as well as purify the reactants to decrease the negative effect of the impurities on enzymes [4]. If enzymes are immobilized on the membrane surface, the substrate enrichment effect due to membrane separation may enhance the enzymatic reaction efficiency [5]. While for the membrane separation, the enzymes immobilized on the membrane surface can change the size or charge pattern of certain solutes and increase the size or charge difference between solutes, thus, improving the separation selectivity by the membrane [5, 6]. On the other hand, the enzymes encapsulated in the membrane can be designed to degrade the pollutants which pass through the skin layer, thereby enhancing the pollutant removal efficiency by the membrane [3]. In addition, biocatalytic membrane enables continuous operation, ameliorates enzyme stability, and facilitates enzyme reuse [7]. Thanks to the above advantages, more and more attentions have been paid on biocatalytic membrane in both academia and industry.

Although biocatalytic membrane upgrades both enzymatic catalysis and membrane separation processes, its commercial or industrial applications are rare because of the following limitations: first, there is a trade‐off between enzyme loading and specific activity in/on the membrane, and increasing enzyme loading normally leads to the decline in membrane permeability and separation selectivity [4]; second, enzyme immobilization by covalent bonding increases the difficulty in enzyme reloading after the immobilized enzymes are inactivated, while noncovalent enzyme immobilization enables enzyme reloading but suffers enzyme leakage during operation [2]; third, membrane fouling and chemical cleaning would inactivate the immobilized enzymes [3]; fourth, the integration of catalysis and separation highly relies on the process control and optimization, and thus, the interface enhancement effect of biocatalytic membrane does not always appear [8]. In order to solve these problems, more efforts need to be made to improve enzyme immobilization methods and design new biocatalytic membranes considering the synergistic effect of catalysis and separation [9]. Moreover, the requirements for enzyme immobilization strategy, membrane property and configuration are different when biocatalytic membranes are applied in various applications. However, most review articles regarding biocatalytic membrane focused on enzyme immobilization techniques [2, 10–12], and few attentions have been paid on the bottlenecks in the applications of biocatalytic membranes. Therefore, this review aims at summarizing the recent progress in preparation of biocatalytic membrane, focusing on mussel‐inspired coating technique and emerging nanomaterials. Then, new advances in the applications of biocatalytic membrane are introduced in brief, and key challenges facing biocatalytic membrane are emphasized and discussed to find possible solutions. This review intends to give help in better understanding intrinsic properties of biocatalytic membrane, and to point out its prospects and research directions.

**PRACTICAL APPLICATION**

Inspired by structure and functions of cell membrane, biocatalytic membrane, which is prepared by immobilizing enzymes in/on a separation membrane, integrates the selective separation ability of membrane and the catalytic activity of enzyme. Biocatalytic membrane not only upgrades both enzymatic catalysis and membrane separation processes, but also enables continuous operation, ameliorates enzyme stability and facilitates enzyme reuse. Protease, glucose oxidase, peroxidase, laccase, carbonic anhydrase, dehydrogenases, β‐galactosidase, dextranase, pectinase and lipase are the most common enzymes for preparing biocatalytic membranes, most of which have been applied for detection, water treatment, biomanufacturing and antifouling. It is found that the desirable configurations of biocatalytic membranes in various applications may be different due to the distinct requirements for separation and catalysis, as well as mass transfer. Therefore, this review article mainly discussed the biocatalytic membrane configurations, and provided directions and guides for the development and industrialization of biocatalytic membrane.

## 2 PREPARATION OF BIOCATALYTIC MEMBRANE

### 2.1 Mussel‐inspired coating for biocatalytic membrane preparation

**FIGURE 2:**
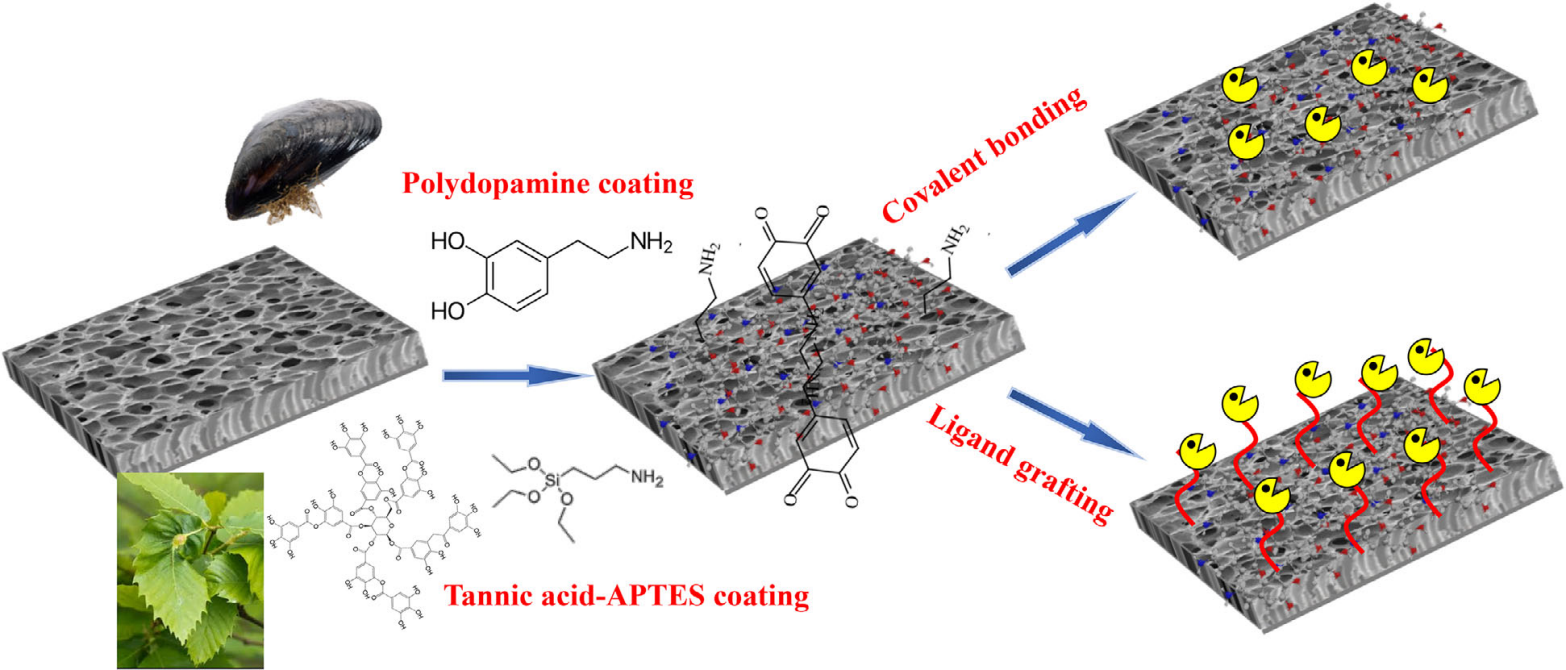
Mussel‐inspired coating for biocatalytic membrane preparation

As shown in Figure 1, enzymes can be immobilized in/on membrane by entrapment, adsorption, covalent bonding, crosslinking, and affinity techniques for preparation of biocatalytic membrane [13, 14]. The physicochemical properties of the membrane are mainly responsible for enzyme loading and activity. In order to increase enzyme loading sites and provide more choices of immobilization strategies, membrane surface activation/modification is a preliminary step for biocatalytic membrane preparation. The traditional methods include ultraviolet or plasma treatment, acid/alkali and organic solvent activation (e.g. glutaraldehyde, 3‐aminopropyltriethoxysilane (APTES), epichlorohydrin, 1‐ethyl‐3‐(3‐dimethylaminopropyl) carbodiimide hydrochloride/*N*‐hydroxysuccinimide, ethylenediamine, trimesoyl chloride). These harsh treatments possibly debase the mechanical stability of the membrane and decrease enzyme activity. Inspired by mussel‐based chemistry [15], Fan et al. activated polyvinylidene fluoride microfiltration (MF) membrane via polydopamine (PDA) coating in aqueous solution (pH 8.5) at room temperature [16, 17]. Since PDA can adhere on various surface by its catechol structure, it offers a chemically and physically versatile platform for further modification and functionalization of membranes, also for preparing biocatalytic membrane [18]. For example, the residual catechol groups on the PDA layer can form covalent bonds with thiol‐ and amino‐containing molecules through Michael addition or Schiff base reaction, and it also can chelate metal ions and coordinate with metal/metal oxide nanoparticles [15]. Thus, two kinds of biocatalytic membranes were constructed by immobilizing laccase via electrostatic and affinity adsorptions, respectively for micropollutant removal [16, 17]. Although rapid and controllable deposition of PDA coating, dopamine‐assisted codeposition, and photoinitiated grafting directly on PDA coating strategies have been developed to improve this coating technology [19], the industrial application of PDA coating is limited because of its high cost and characteristically dark color. Tannic acid (TA), as a green and low‐cost plant‐based polyphenol, also can form an active coating layer on diverse materials but the stability is questioned. Based on mussel‐inspired catecholamine chemistry, the catechol−amine codeposition can form polyphenol/polyamine oligomers via the Michael addition, which would significantly enhance the stability of the coating. As illustrated in Figure. 2, Zhou et al. established a TA−APTES coating for membrane activation and subsequent enzyme loading via covalent bonding, and secondary grafting branched polymer could further increase enzyme loading [20]. Thanks to the hierarchical nanostructure and abundant quinone groups of the TA–APTES coating, the enzyme loading and specific activity on the biocatalytic membranes prepared by TA–APTES coating outperformed the PDA‐activated ones [20]. Furthermore, TA–APTES coating is able to increase the membrane surface hydrophilicity and antifouling performance. Therefore, such an advanced coating strategy has great potential in biocatalytic membrane preparation at large scale.

### 2.2 Emerging nanomaterials for biocatalytic membrane preparation

Due to the limited specific area of the membrane surface, the low enzyme loading on the membrane leads to a weak catalytic efficiency of the biocatalytic membrane, restricting its applications under flow‐through mode (short retention time of substrate). In order to increase enzyme loading, some emerging nanomaterials, such as magnetic nanoparticles, carbon nanotubes (CNTs), graphene oxides (GO), and metal organic frameworks (MOFs), have been introduced into the membrane. Gebreyohannes et al. utilized superparamagnetic nanoparticles as enzyme carrier and as nanofiller to form organic–inorganic hybrid membrane, which not only increased surface area for enzyme immobilization but also achieved full recovery and reuse of the enzyme under reversible magnetic force [21]. Ji et al. prepared a stable CNTs coating on a polymeric membrane for laccase immobilization via both physical adsorption and covalent bonding, and the fresh laccase could be reloaded onto the coating layer by adsorption after the inactivated enzymes were washed away by surfactant solution [22]. Zhang et al. first embedded carbonic anhydrase (CA) into MOFs to enhance enzyme stability, and then fabricated a biocatalytic membrane via situ growth of MOFs@CA nanocrystal seeds on a modified polymeric membrane, showing a significant improvement in CO_2_/N_2_ selectivity compared to the membrane without the embedded CA [23]. In these studies, the nanomaterials with immobilized enzymes are deposited on the membrane surface, inevitably increasing the filtration resistance. For an asymmetric membrane, normally it has a porous nonwoven fabrics support, which owns a large space for loading nanomaterials without a sacrifice of permeability. Ren et al. first reversed membrane surface charges by polyethyleneimine coating for immobilizing laccase mainly by electrostatic adsorption, and then the water‐stable MOFs particles were adhered on the support layer fibers by reverse filtration and PDA coating, which could broaden enzyme distribution in the membrane, enhance the membrane adsorption capacity to laccase and micropollutant, thus, improving membrane permeability, reducing enzyme leakage, enhancing pH tolerance, and reusability of the biocatalytic membrane for micropollutants removal [24]. Moreover, MOFs can be designed as artificial substitutes of enzymes, ameliorating the stability of the biocatalytic membrane [25]. On the other hand, nanomaterials, such as GO, is able to promote enzyme activity by their substrate enrichment effect and catalytic functions. Zhang et al. inserted GO and GO derivates into a biocatalytic membrane by reverse filtration and codeposition methods respectively, which increased the enzyme loading and its storage stability [26]. However, since GO in the membrane also absorbed polymerized products and resulted in more serious membrane fouling, the GO‐based biocatalytic membranes showed lower micropollutant removal efficiency and negligible improvement in reusability. Therefore, the strong adsorption ability of nanomaterials may be a “double‐edged sword” for biocatalytic membrane if the catalytic products are less hydrophilic [27]. While integrating the catalysis and carrier functions of nanomaterials may be promising to prepare the biocatalytic membrane for cascade reactions (chemo‐ and biocatalytic functionalities in a single nanostructure). For example, Dutta et al. designed a nanoreactor with coordinatively unsaturated metal cations, heterogeneous metal nanocrystals, and enzyme catalysts within a single mesoporous MOF for multistep cascade reactions [28], which gives inspiration for biocatalytic membrane preparation.

## 3 APPLICATIONS OF BIOCATALYTIC MEMBRANE

Protease, glucose oxidase, peroxidase, laccase, CA, formate dehydrogenase, formaldehyde dehydrogenase, alcohol dehydrogenase, β‐galactosidase, dextranase, pectinase, and lipase are the most common enzymes for preparing biocatalytic membranes, most of which have been applied for detection, water treatment, biomanufacturing, and antifouling. It is found that the desirable configurations of biocatalytic membranes in various applications may be different due to the distinct requirements for separation and catalysis, as well as mass transfer.

### 3.1 Bioassay and biosensor

Biocatalytic membranes have been widely investigated for protein digestion and glucose detection [29]. Proteolysis is a preliminary step in most analyses of proteins by mass spectrometry. Compared with traditional in‐solution digestion, the biocatalytic membrane with immobilized protease for rapid protein digestion under flow‐through mode can increase throughput and facilitate online mass spectrometry analysis. These advantages of biocatalytic membrane are attributed to its high enzyme concentration on the liquid–solid interface and superior substrate mass transfer by convection. Moreover, by easily and precisely controlling the residence time of protein in the biocatalytic membrane at different flow rates, the obtained peptide lengths can be regulated to increase the sequence coverage of peptides [30]. For protein digestion, the enzyme carriers used are normally MF membranes, which only enable enzyme immobilization and accelerate mass transfer, without exploiting their selective separation function. While regarding glucose detection in blood, biocatalytic membrane is supposed to separate blood proteins from glucose for avoiding interference and to conduct electrons for sensing/amplifying signals, thus, a more complex membrane configuration with special materials is required. Chu et al. designed a novel Prussian Blue‐based biocatalytic membrane owing a separation layer with dense micropores and a sensing layer with loose macropores, which could achieve blood separation and detection synchronously [31]. The separation layer only allowed serum to enter the internal channels via sieving effect and to contact the glucose oxidase‐immobilized sensing layer, while the Prussian Blue sensing layer with high conductivity could effectively transfer electrons to generate a sensitive response signal. Huang et al. designed a flexible biocatalytic membrane with excellent stability and electrochemical performance for implanted glucose monitoring, which was constructed by the coimmobilization of the glucose oxidase microparticles and multiwall CNTs on the inner surface of a gradient‐structured hollow fiber membrane, where CNTs enhanced the electron transfer efficiency and the membrane separated the interferents from glucose [32]. Since bioassay/biosensor market is not so sensitive to the cost, biocatalytic membrane has great potential to be commercialized in the healthcare fields.

### 3.2 Organic micropollutants removal

**FIGURE 3:**
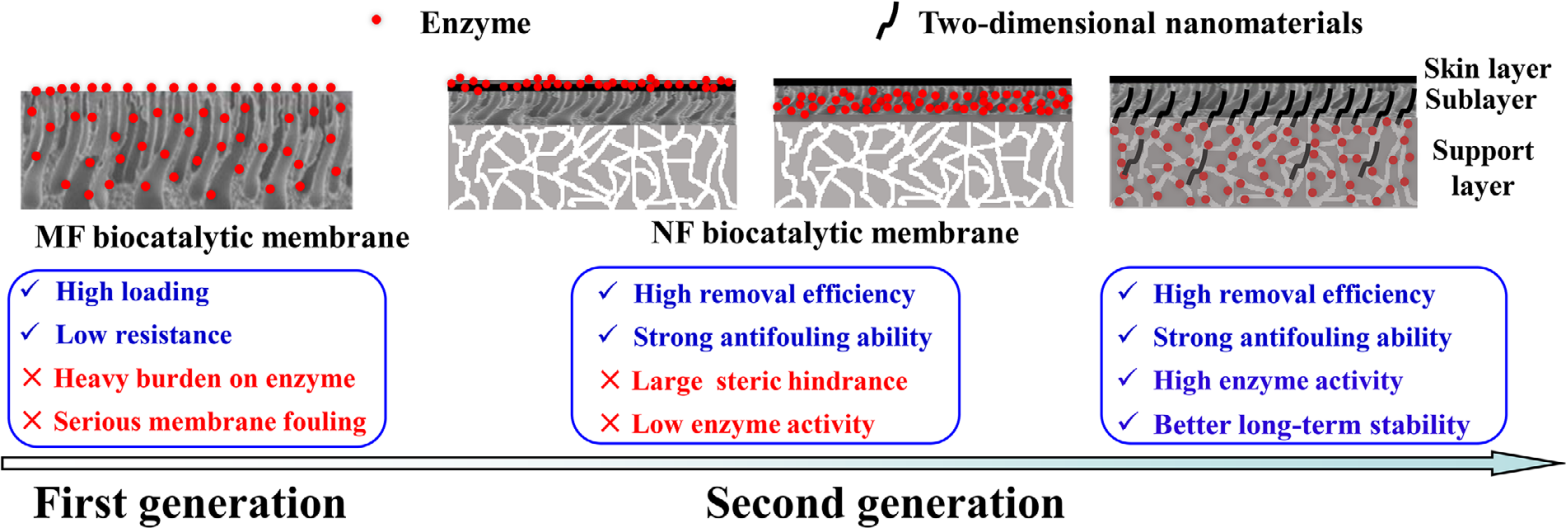
Biocatalytic membranes with different configurations for micropollutant removal

Organic micropollutants, as a kind of small molecule with trace concentrations in water, such as antibiotics, endocrine disrupting compounds, pharmaceuticals and personal care products, are difficult to degrade and detrimental to human. Some oxidases (e.g. peroxidase, tyrosinase, laccase) can oxidize and polymerize these micropollutants, greatly reducing their toxicity. In order to increase the enzyme stability, modified polymeric or ceramic MF membranes are widely used as carriers for enzyme immobilization via physical adsorption or covalent bonding, which significantly improve the enzyme reusability during the micropollutant removal [16, 17, 22, 33, 34]. As shown in Figure 3, although enzyme loading is high and filtration resistance is low, the hydrophobic polymerized products and other foulants are prone to adsorb in the MF membrane, resulting in serious fouling and enzyme inactivation [16]. Li et al found that when laccase was immobilized on a nanofiltration (NF) membrane, the polymerized products would be fully retained by the modified NF membrane with super high hydrophilicity, leading to a high and stable bisphenol A (BPA) removal [5]. However, the laccase activity on the membrane was weak due to the low enzyme loading and high operating pressure, and it was required to add mediators into the membrane system for accelerating electron transfer and promoting enzyme activity on BPA. Laccase can also be immobilized in the NF membrane by reverse filtration and subsequent PDA coating (for sealing the enzyme), and Cao et al. confirmed that a high BPA removal by such a biocatalytic membrane could be obtained thanks to a combination of separation (reducing the enzymatic burden), adsorption (enriching the substrate concentration as well as prolonging the residence time) and finally, catalysis (oxidizing the pollutants and breaking the “adsorption saturation limits”) [3]. However, the polymerized products would be retained by the sealing layer and accumulated in the membrane, gradually decreasing enzyme activity. This problem may be solved if enzymes are stably immobilized in the support layer without the sealing layer which ensures the products freely passing through the membrane. Moreover, inserting two‐dimensional nanomaterials (e.g. graphene and derivatives, transition‐metal oxides, layered double hydroxides, transition‐metal dichalcogenides, MXenes) into the membrane possibly increases permeability and enzyme activity (Figure 3, right).

### 3.3 Carbon dioxide capture and conversion

**FIGURE 4:**
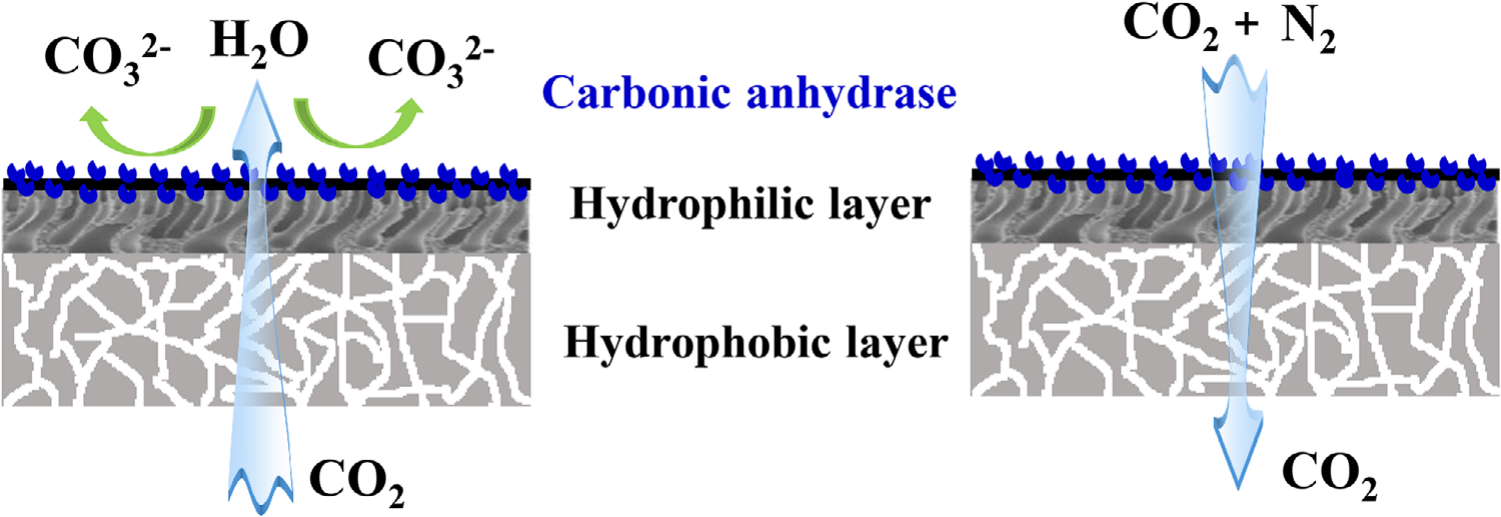
Janus biocatalytic membrane for CO_2_ capture and separation

Carbon dioxide (CO_2_) capture by CA is a hot topic in recent years and the immobilization of CA has been widely studied. As the enzymatic reaction for the immobilized CA happens at the gas–liquid–solid interface, Janus biocatalytic membrane with different hydrophobicities is desirable for CO_2_ capture. As illustrated in Figure 4 (left), Hou et al. constructed a Janus biocatalytic membrane with a hydrophilic CNTs layer coated on a fluorosilane treated superhydrophobic MF membrane, and CA was immobilized on the hydrophilic CNT side (CO_2_‐solvent interface), while the superhydrophobic porous side of the membrane was facing the gas phase [35]. Such a “Janus” configuration ensured the immobilized CA remaining hydrated and minimized the CO_2_ diffusion path in the solvent, thus, improving the CO_2_ capture efficiency. Janus biocatalytic membrane can also be used for CO_2_ separation. As shown in Figure 3 (right), Fu et al. prepared a Janus membrane with a ∼18‐nm‐thick close‐packed array of 8 nm diameter hydrophilic pores that stabilized water by capillary condensation and precisely accommodated CA, and a 50‐μm‐thick hydrophobic support could prevent liquid passing through the membrane [36]. The highly concentrated CA in the hydrophilic layer catalyzed the rapid interconversion of CO_2_ and water into carbonic acid, thus, accelerating the CO_2_ permeation through the membrane and improving CO_2_/N_2_ and CO_2_/H_2_ selectivity. On the other hand, conversion of CO_2_ into methanol by three dehydrogenases simultaneously reduces greenhouse gases and produces fuels. Luo et al. firstly coimmobilized formate dehydrogenase, formaldehyde dehydrogenase, and alcohol dehydrogenase in a polymeric membrane by reverse filtration, and the resultant biocatalytic membrane achieved the bioconversion of CO_2_ to methanol (0.5 mM) [8]. Using a suitable ionic liquid as buffer solution, methanol yield increased by 3.5‐fold due to higher CO_2_ solubility in the biocatalytic membrane system [37]. Zhu et al. applied MOFs to entrap the dehydrogenase and coenzyme, and three kinds of the obtained nanocomposites were embedded into different MF membranes for enhancing CO_2_ conversion [38]. However, it can be concluded that for CO_2_ capture and conversion by biocatalytic membrane, all the studies are only for proof concept and far from the practical application.

### 3.4 Biomolecules production

Biocatalytic membrane with lipase has been widely used to construct biphasic enzymatic membrane reactor for production of biodiesel and pharmaceuticals [1, 2]. Wang et al. prepared a Janus ultrafiltration (UF) membrane with hydrophilic cellulose acetate layer and hydrophobic polytetrafluoroethylene layer for lipase immobilization by simple filtration, which showed not only higher enzyme stability but also better chiral selectivity in the ibuprofen production [39]. This noncovalent immobilization strategy (entrapment of enzymes in the membrane by reverse filtration) can realize super high enzyme loading and well retain enzyme activity [14, 40, 41], and it has also been used for organic acid conversion (e.g. l‐malic acid) with hollow fiber membrane [7]. Moreover, biocatalytic membrane can control the molecular weight of product by its separation function. For example, Raaijmakers et al.fabricated a biocatalytic UF membrane via facile interfacial polycondensation of pepsin and trimesoyl chloride on a porous support, and the pore size of this membrane could be adjusted by changing trimesoyl chloride concentration in order to obtain the desirable peptide size in the permeate [42]. However, normally the pore size distribution of the membrane is not uniform, resulting in an uneven molecular weight product. Su et al. reported that the prefiltration of dextranase to form a fouling layer on the UF membrane could not only stabilize the enzyme but also narrow the membrane pore size distribution, thus, leading to a higher uniformity of oligodextran products [43]. Inspired by this work, it is noted that during the preparation of biocatalytic membranes for biomolecule production, the effect of enzyme immobilization on membrane pore size and separation selectivity needs to be considered.

### 3.5 Antifouling enhancement

**FIGURE 5:**
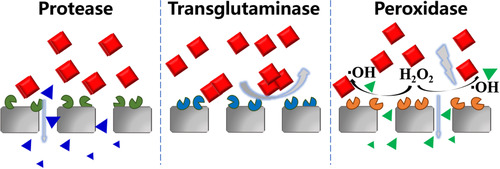
Biocatalytic membrane with different enzymes for mitigating protein fouling

Since enzyme can degrade specific molecules, biocatalytic membrane has also applied for antifouling enhancement. For instance, Vanangamudi et al. immobilized trypsin and α‐chymotrypsin on a UF membrane via direct covalent binding, and the resultant biocatalytic membranes had stable protein antiadhesion and self‐cleaning abilities because of the repulsive mechanism and digestion of proteins into peptides/amino acids (Figure 5, left) [44]. Besides protein degradation, proteins cross‐linking by transglutaminase can also reduce membrane fouling. Wang et al. prepared a biocatalytic membrane with transglutaminase by covalent bonding, and found that during whey treatment, the filtration resistance of the biocatalytic membrane was approximately 50% less than that of pristine polyethersulfone membrane mainly due to higher shear‐induced back diffusion of the cross‐linked proteins [45] (Figure 5, middle). Although such a biocatalytic membrane can efficiently degrade or cross‐link one specific foulant, it would be invalid when facing complex fouling. Therefore, a biocatalytic membrane with peroxidase may be universally effective on different foulants by free radicals’ attack, and the free radicals would be produced by peroxidase catalyzing hydrogen peroxide added (Figure 5, right). Moreover, Kim et al. stated that a biocatalytic membrane with acylase maintained 66% of its initial enzyme activity for 200 days under rigorous shaking, obviously inhibiting the biofouling formation on the membrane via quorum quenching [46].

## 4 KEY CHALLENGES FACED BY BIOCATALYTIC MEMBRANE

Although biocatalytic membrane has been studied in various applications, its scale‐up and commercialization are difficult and still in progress because of the following challenges.

### 4.1 Trade‐off between enzyme activity and stability

It is well known that noncovalent immobilization well retains enzyme activity in/on the membrane but suffers enzyme leakage, while covalent bonding undergoes the reverse effects. Entrapment of enzyme in the membrane seems to solve this dilemma [7], but if the impurities or products can also be entrapped into the membrane, the stability of the biocatalytic membrane would be questionable due to irreversible fouling formation [47]. Cao et al. found the density of sealing layer had a significant effect on the enzyme activity and operating stability, and a sealing layer with higher density resulted in less enzyme leakage but lower enzyme activity and worse operating stability due to the larger mass transfer resistance of both substrate and polymerized product [48]. Thus, the combination of noncovalent adsorption and entrapment mechanisms for tightly immobilizing enzyme in a membrane with a porous structure allowing free permeation of substrate and products may realize both high activity and stability [48]. On the other hand, a mild membrane activation strategy for direct covalent bonding of enzymes by biocompatible molecules, such as the catechol−amine codeposition in aqueous solution, may break the trade‐off between enzyme activity and stability on the membrane [20].

### 4.2 Coimmobilization of enzymes/coenzymes in/on membrane

Many enzymatic reactions involve several enzymes/coenzymes, and thus, coimmobilization of enzymes/coenzymes in/on the membrane is required. However, due to the low specific area on the membrane, immobilizing enzymes/coenzymes on the membrane results in low loading and limited activity. Hence, the membrane pores and support layer with a high specific area or a large space are more suitable for coimmobilization. Li et al. prepared a biocatalytic membrane with two enzymes covalently binding in a porous aluminum oxide membrane with a pore diameter of 90 nm, which accelerated enzymatic reactions by minimizing the diffusion loss of intermediate species compared with free enzymes [49]. Entrapment of several enzymes/coenzymes in the UF membrane by reverse filtration can also achieve a high enzyme loading/activity and an enhanced conversion of CO_2_ [8, 38], but the resulting membrane permeability loss (larger filtration resistance and limited mass transfer) may be problematic for a liquid substrate [50]. Porous nanomaterials as both chemical catalyst and enzyme carrier can be embedded into membrane for cascade reaction, which may greatly alleviate such permeability loss.

### 4.3 Trade‐off between enzyme loading and membrane permeability

Higher enzyme loading in the membrane pores definitely causes a lower permeability, and especially under reverse filtration mode, more and more enzymes would accumulate and be compacted in the pores, leading to decreasing productivity. If higher pressure is applied to increase the permeate flux, it may have negative effects on the enzyme activity [5]. Zhang et al. immobilized glucose oxidase and HRP in a NF membrane by reverse filtration and then sealed the enzymes in the membrane by PDA codeposition, and the enzymes were tightly immobilized by multimechanisms, such as adsorption, entrapment, and covalent bonding; most importantly, such a biocatalytic membrane could be operated in normal mode (separation layer facing the feed) and the enzymes would not accumulate in the pores, thus keeping a high membrane permeability during long‐term operation [51]. Moreover, as shown in Figure 3 (right), enzymes may be immobilized on the support layer fibers, thus, increasing enzyme loading without a sacrifice of permeability.

### 4.4 Substrate accessibility to immobilized enzymes

When enzymes are immobilized in the membrane, the accessibility of large substrate to the immobilized enzymes becomes a problem. Thus, enzyme immobilization on the membrane surface is necessary for the large substrate. In order to further increase the substrate accessibility to the immobilized enzymes on the membrane, spacer arms can be firstly grafted on the membrane to enhance the flexibility of the immobilized enzyme [18], and a branched polymer as spacer arm may also magnify enzyme loading [20]. On the other hand, microspheres with immobilized enzymes on their surface can be deposited at the membrane surface and form a dynamic catalytic layer for initial degradation of large substrate, and then the small intermediates enter biocatalytic membrane for further reaction [52]. This special biocatalytic membrane with a multilayer construction (also called microsphere‐membrane integrated enzymatic reactor) is promising for peptide and oligosaccharide production.

### 4.5 *“*In‐situ*”* product removal and product molecular weight distribution

The greatest advantage of biocatalytic membrane is the *“*in‐situ*”* product removal ability during enzymatic reaction for reducing product inhibition and avoiding byproduct generation, which is more beneficial for large molecules degradation (e.g. peptide and oligosaccharide production) because the product molecular weight can be controlled by optimizing permeate flux (i.e. retention time) [42]. However, the product molecular weight distribution largely depends on the membrane properties, concentration polarization, and fouling layer formation. Since the pore size distribution of the commercially available membranes is uneven, it is not easy to get high‐quality product with narrow molecular weight distribution. As mentioned in Section 3.4, enzyme immobilization can also be used for regulation of membrane pore size and its distribution, improving the product molecular weight distribution [43].

### 4.6 Membrane fouling

Membrane fouling is a ubiquitous problem for all the membrane technologies, which is particularly important for biocatalytic membrane because the fouling would not only deteriorate separation performance but also inactivate enzymes. In order to alleviate membrane fouling of biocatalytic membrane, a pure substrate solution is recommended. Even so, the fouling layer formed by products and intermediates also needs to be considered, and normally it can be reduced by optimizing membrane materials (less adsorption) and pore size (less pore blocking). Moreover, changing the enzymatic reaction pathway may decrease the product fouling. For example, the biocatalytic membrane with laccase suffers the fouling of polymerized products, and Zhang et al. replaced laccase by glucose oxidase and HRP for the preparation of biocatalytic membrane [51]. The cascade catalysis by these two enzymes could be well‐tuned by changing the bulk glucose concentration and permeate flux, thus, achieving the efficient oxidation of BPA and at the same time avoiding the polymerized product formation.

### 4.7 Membrane cleaning and regeneration of biocatalytic membrane

When the fouling of biocatalytic membrane greatly affects the separation and catalytic performance, a mild cleaning strategy is required to remove foulants and recover enzyme activity. Cao et al. found that after the fouled membrane was immersed in 50% ethanol for 30 s and then washed by ultrapure water for 5 min, the polymerized product of BPA could be removed, but enzyme activity also decreased with cleaning cycles [3]. Since most chemical cleaning agents are harmful to enzyme configuration and activity, the regeneration of biocatalytic membrane by reloading fresh enzymes is another option to reuse the membrane. The biocatalytic membrane prepared by reversible enzyme immobilization strategies, such as adsorption and affinity methods, can be regenerated by simple elution and reloading [5, 53, 54]. As mentioned in Section 4.1, enzyme leakage would occur when enzymes are immobilized by adsorption, but the leaked enzymes may be recaptured by the ligands on the membrane if the biocatalytic membrane itself can fully retain the enzymes [5]. Moreover, Marpani et al. constructed a porous gel layer for encapsulation of enzymes on a PDA‐coated UF membrane via pressure‐driven filtration of alginate, enzyme, calcium, and PEG solution, and the inactivated gel layer could be dissolving by hot water cleaning for remaking a new active layer [55].

## 5 CONCLUDING REMARKS

A biocatalytic membrane with high enzyme loading/activity, high permeability, and separation performance as well as good long‐term storage/operation stability is desirable for real applications. Besides enzyme immobilization, the surface/interior property and configuration of biocatalytic membrane are important to its performance, and these requirements are different for various applications. For large substrate, enzymes can be covalently linked with a spacer arm which is grafted on a catechol−amine coated membrane, and a microsphere‐membrane integrated enzymatic reactor is also preferred. While for small substrate, enzymes may be stably immobilized in the membrane by multimechanisms allowing the product freely passing through the membrane. Although introducing nanomaterials into membrane can increase enzyme loading and may enhance catalytic activity, their adsorption to products deteriorating the membrane stability should be avoided. Biocatalytic membrane has great potential in the applications of online detection, macromolecule degradation, and small molecule conversion. In order to achieve the reuse of biocatalytic membrane, the purification of substrate is essential, or the enzyme can be reloaded via reversible enzyme immobilization techniques because chemical cleaning for removing membrane fouling inevitably causes enzyme inactivation. To facilitate the application of biocatalytic membrane in industry, the membrane materials, pore size, configuration, and operating mode should be rationally designed for a specific purpose, and then a suitable enzyme immobilization strategy is matched for maximizing the synergistic effect of catalysis and separation.

## CONFLICT OF INTEREST

The authors have declared no conflict of interest.
